# Visible colorimetric growth indicators of *Neisseria gonorrhoeae* for low-cost diagnostic applications

**DOI:** 10.1371/journal.pone.0252961

**Published:** 2021-06-17

**Authors:** Taylor Mae Oeschger, David Carl Erickson

**Affiliations:** 1 Meinig School of Biomedical Engineering, Cornell University, Ithaca, New York, United States of America; 2 Sibley School of Mechanical and Aerospace Engineering, Cornell University, Ithaca, New York, United States of America; 3 Division of Nutritional Science, Cornell University, Ithaca, New York, United States of America; Instituto Butantan, BRAZIL

## Abstract

*N*. *gonorrhoeae* is one of the most pressing antibiotic resistant threats of our time and low-cost diagnostics that can easily identify antibiotic resistance are desperately needed. However, *N*. *gonorrhoeae* responds so uniquely to growth conditions that it cannot be assumed gonorrhea will respond to common microbiological methods used for other pathogenic organisms. In this paper, we explore visual colorimetric indicators of *N*. *gonorrhoeae* growth that can be seen without a microscope or spectrophotometer. We evaluate growth media, pH indicators, resazurin-based dyes, and tetrazolium-based dyes for their use in simple colorimetric system. Overall, we identified Graver Wade media as the best at supporting robust gonococcal growth while also providing the least background when analyzing results of colorimetric tests. XTT, a tetrazolium-based dye, proved to show to brightest color change over time and not negatively impact the natural growth of *N*. *gonorrhoeae*. However, other dyes including PrestoBlue, MTT, and NBT are less expensive than XTT and work well when added after bacterial growth has already occurred. By identifying the specific use cases of these dyes, this research lays the groundwork for future development of a color-based antibiotic susceptibility low-cost test for *N*. *gonorrhoeae*.

## Introduction

The rapidly developing multi-drug resistance of *Neisseria gonorrhoeae* has been identified as a major public health threat by the World Health Organization [1], the National Institute of Health [2], and the Centers for Disease Control [3]. For *N*. *gonorrhoeae*, phenotypic antibiotic susceptibility testing remains standard due largely to the cost of genotypic testing and the vast array of genes involved in resistance [4,5]. Low-cost and point-of-care diagnostics for identification of *N*. *gonorrhoeae* infections have increased the number of patients treated compared to symptomatic management alone [6,7], however, increased treatment without careful consideration of specific antibiotics used may drive further antibiotic resistance in this organism [8]. Therefore, many experts have called for the creation of a low-cost test that enables gonococcal antimicrobial resistance monitoring worldwide [9–12]. Modeling studies have demonstrated that an antimicrobial resistance point-of-care test for gonorrhea could drastically delay the onset of untreatable gonorrhea by 10 years or more [13–17].

*N*. *gonorrhoeae* is a notoriously fastidious, obligate human pathogen with limited clinically relevant models that does not grow well in standard liquid medias [18]. Previous research has shown that even the widely used, non-toxic resazurin dye inhibits *N*. *gonorrhoeae* growth so significantly that it could be used a treatment option [19]. Therefore, it is important for growth media and colorimetric indicator combinations to be specifically tested on this unique organism. The ideal colorimetric indicator system should be: 1) visible to the naked eye, 2) low cost, 3) not inhibit the natural growth of bacteria, and 4) display minimal strain-to-strain variability. Herein, we evaluate three categories of dyes: pH indicators, resazurin indicators, and tetrazolium salts. These dyes were selected for qualities related to low-cost applications such as their applicability to suspended cell cultures, ease of use, and low cost.

pH indicators are one of the oldest colorimetric indicators and are often included in commercial medias, but until now their usefulness has not been evaluated for studying *N*. *gonorrhoeae*. Meanwhile, resazurin is a redox dye that is reduced by bacteria to form a differently-colored product [20]. While many forms and protocols for resazurin exist, we selected the commercially modified PrestoBlue which advertises faster reduction times compared to resazurin salt. Similarly, tetrazolium salts have many forms and multiple generations, each with unique chemical properties. From commonly used tetrazolium-based dyes, we selected two first-generation salts, MTT and NBT, which form insoluble formazan and one second-generation salt, XTT, which forms a soluble formazan, all reactions that produce a visual color change. We used the Clinical and Laboratory Standard Institute’s recommend clinical control strain *N*. *gonorrhoeae* ATCC 49226 [21,22] and the WHO Neisseria Reference panel from the Centers for Disease Control and Food & Drug Administration’s Antibiotic Isolate Bank [23,24] to test the various dye and media pairs.

## Results

### Media selection

**Fig 1:**
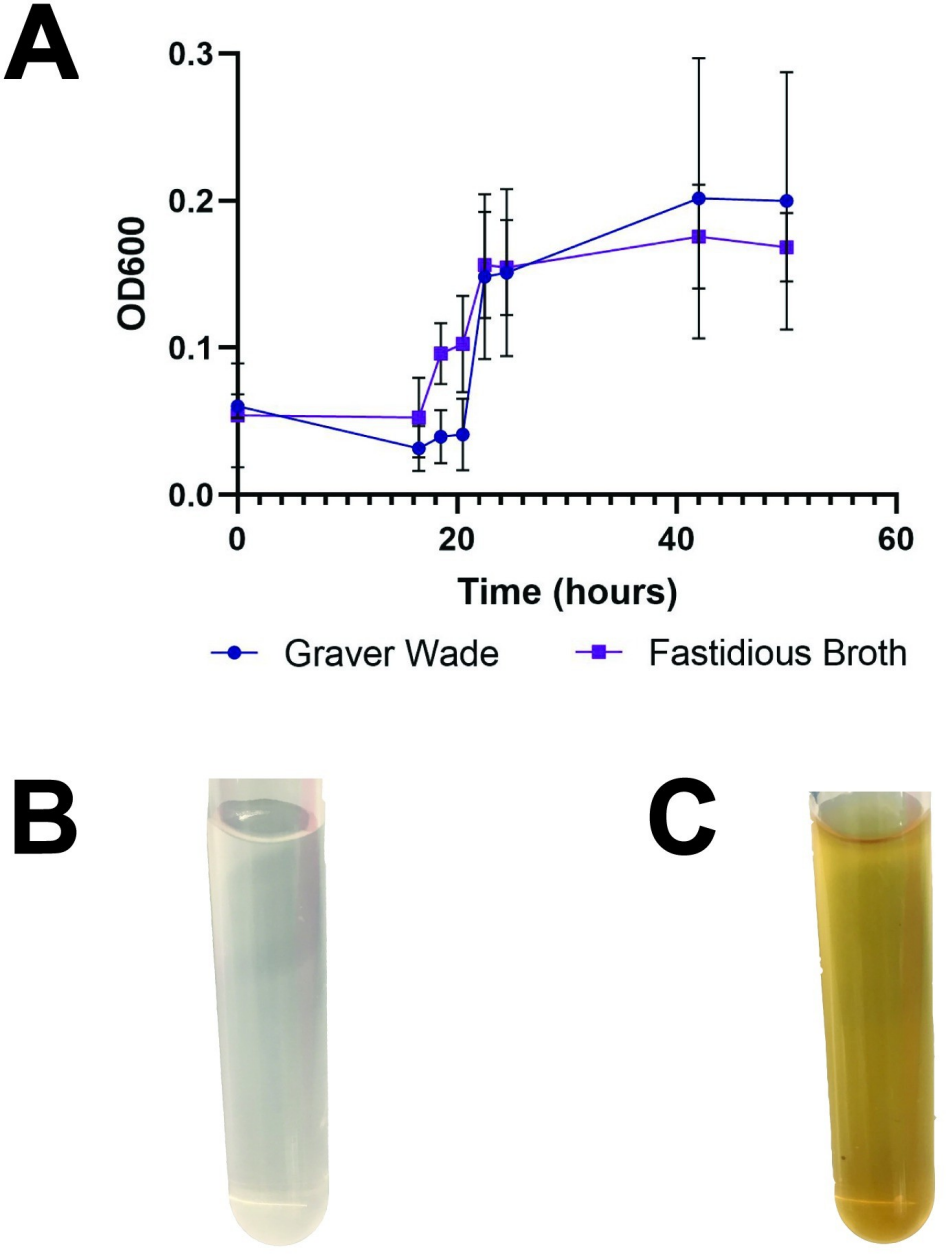
Liquid growth medias. (A) Growth curve of N. gonorrhoeae ATCC 49226 in Graver Wade (circles) and Fastidious Broth (squares). (B) 24-hour culture tube of Graver Wade and (C) 24-hour culture tube of Fastidious Broth.

*N*. *gonorrhoeae* struggles to grow in liquid media since it is a bacterium of mucosal surfaces and requires CO_2_ to initiate growth. Therefore, higher inoculating concentrations are required [25]. Since there is no clinically defined standard liquid media, we compared the two most robust liquid gonococcal growth medias used in previous studies: Fastidious Broth and Graver Wade Media [26]. Over 48 hours of growth, both medias were able to support growth of *N*. *gonorrhoeae* strain ATCC 49226 as measured by spectrophotometry and results were average across 3 replicates (Fig 1A). The difference between the two medias was not statistically significant (p = 0.402). Both medias have previously been validated in a variety of *N*. *gonorrhoeae* strains [26–28] but these medias have never been specifically examined in the context of colorimetric growth indication. While the two medias offered similar growth support, Graver Wade media is clear and colorless (Fig 1B) compared to the dark yellow appearance of Fastidious Broth (Fig 1C). However, Fastidious Broth is commercially available in a ready-to-use format, whereas Graver Wade must be measured, mixed, and sterilized by the user. Therefore, the designer of low-cost technology relying on liquid culture of *N*. *gonorrhoeae* must prioritize either media color or ease of media acquisition when selecting between these options. For our studies, we chose to use Graver Wade in future experimentation so we could visualize the true color of the dyes.

### pH indicators

pH indicators medias are often added to commercial agar or liquid media as an indicator of growth or metabolic function. When bacteria are grown in weakly buffered solutions, they release acidic waste products during growth and replication that change the pH of the solution. Thus, a pH indicator that changes color between the starting pH and the final pH of the media can be used to signal that acidic products have been release by viable bacteria [29]. pH indicators have been used in food and environmental monitoring for this purpose [29–31]. For gonorrhea specifically, pH indicators have been shown to detect the presence of penicillinase release by some *N*. *gonorrhoeae* strains [32], but the use of pH indicators as a broader viability marker has never been explored.

**Fig 2:**
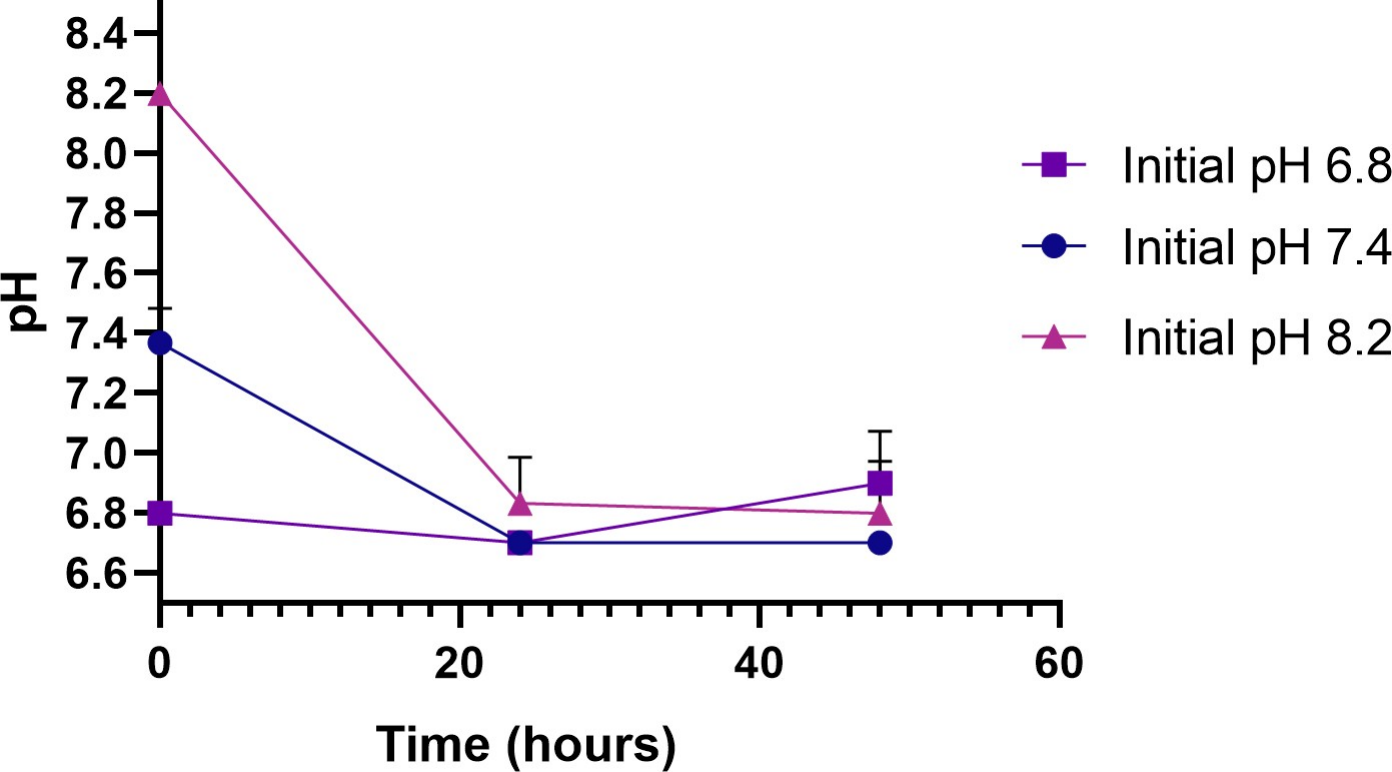
pH during culture. pH over 48-hour Cultures of *N*. *gonorrhoeae* ATCC 49226.

Like many microorganisms, *N*. *gonorrhoeae* prefers a near neutral pH, specifically a pH of approximately 6.8. For this reason, Graver Wade media is adjusted to a pH of 6.8 prior to inoculation to support ideal growth, however, we found that *N*. *gonorrhoeae* will grow at more basic pH, up to at least 8.2, and release acidic products that reduce the pH back to the preferred 6.8 (Fig 2). While it is theoretically possible to use a pH indicator that changes color between 8.2 and 6.8, those typically used in microbiology such as bromothymol blue, methyl red, and phenol red are not sensitive enough in this range to detect low levels of *N*. *gonorrhoeae* growth needed for clinical diagnostics. Additionally, adjusting the pH away from ideal growth could inhibit initial growth and therefore impact clinical results. Therefore, pH indicators are not an ideal choice as a low-cost gonococcal growth indicator.

### Viability dyes

#### Resazurin-based dye

**Fig 3:**
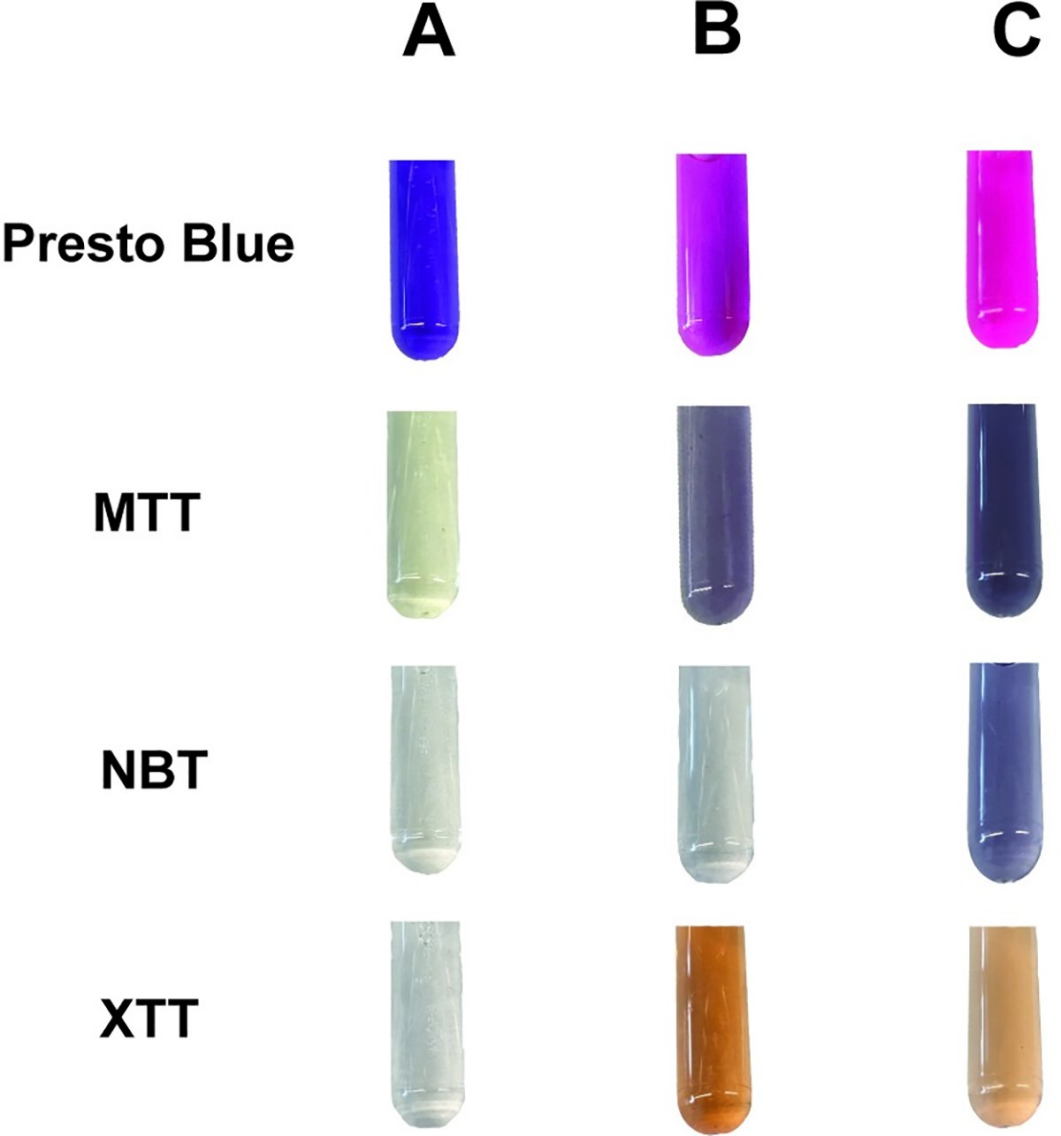
PrestoBlue, MTT, NBT, XTT during and after culture. N. gonorrhoeae ATCC Clinical Control Strain 49226 in 1/10 PrestoBlue, 0.1mg/mL MTT, 0.1mg/mL NBT, and 0.1mg/mL XTT: (A) At time 0, (B) At time 24hr with dye added at time 0, and (C) At time 24hr with dye added at 23hr.

PrestoBlue (ThermoFischer Scientific, USA) is a proprietary, ready to use, lysis-free, resazurin-based cell viability dye. The purple-colored resazurin (Fig 3A) is reduced by metabolic products of bacterial cells to produce pink-colored resorufin (Fig 3B and 3C), which can be further reduced to hydroresorufin, which is clear and colorless [33]. This color change is fluorescent at a excitation wavelength of 530-570nm with emissions at 585-590nm wavelength [34], but changes are also easily seen by eye [35], making it applicable to a variety of low-cost technologies [36–38]. Resazurin is typically diluted to a working concentration of ~0.1mg/mL [39], but for PrestoBlue, the manufacturer recommends a standard 10% v/v dilution of the stock solution to obtain a working concentration. PrestoBlue is pH buffered and is reduced in as little as 10 minutes, significantly shorter than resazurin alone [40].

When *N*. *gonorrhoeae* is incubated for 24 hours with 10% v/v PrestoBlue little color change is seen (Fig 3B) compared to adding 10% v/v at hour 23 and allowing an additional hour for bacterial growth and dye reduction (Fig 3C). As these cultures are otherwise identical, this implies that PrestoBlue is inhibiting the metabolism of *N*. *gonorrhoeae*, an unfortunate side effect of many viability dyes. Indeed, this is confirmed by Schmitt *et al*. [19] who determined that pure resazurin exhibits antimicrobial activity against a broad range of *N*. *gonorrhoeae* strains, even at only 2.8μg/mL. Even the reduced form, resorfurin pentyl ether, inhibited *N*. *gonorrhoeae* growth, although with a higher minimum inhibitory concentration [19]. This result is surprising since resazurin is generally considered to be non-toxic [40], however, *N*. *gonorrhoeae* and *Francisella tularensis* both possess unique lipoprotein sorting machinery which resazurin may block [19,41]. Regardless, resazurin-based assays for *N*. *gonorrhoeae* have been developed, with resazurin added at the end of growth [39] and no variation was found between the colors of the dye post growth (S1 Fig). The exact concentration of pure resazurin in PrestoBlue is unknown, however, it still appears to display reliable color change in the presence of a 24-hour gonococcal culture, implying that the toxic effects of PrestoBlue may be less than that of resazurin alone, although further experimentation is needed to confirm this.

#### Tetrazolium-based dyes

MTT, NBT, and XTT are tetrazolium salts that are reduced to visibly colorful formazans by cells during growth. MTT and NBT are first-generation tetrazolium salts that are pulled across the cell membrane due to their net positive charge and form insoluble formazans inside bacteria [42]. XTT on the other hand is a second-generation tetrazolium salt with a negative charge that requires an electron acceptor to be completely reduced to form a soluble formazan [43]. Most research on tetrazolium salts has been primarily performed on mammalian cultures [44–46], but they are broadly applicable for bacterial cultures as well [47–49]. Here we evaluated MTT, NBT, and XTT for their use in identifying *N*. *gonorrhoeae* growth rapidly and consistently.

MTT at a working concentration of 0.1mg/mL appears pale yellow before growth (Fig 3A). When added at the beginning of 24 hours of *N*. *gonorrhoeae* growth, it turns a grey purple (Fig 3B). This color is more distinct when the dye is added after 23 hours of growth and incubated for an additional hour (Fig 3C). This implies that MTT has some inhibitory effect on the *N*. *gonorrhoeae* growth, which is not surprising since some tetrazolium dyes display microbial toxicity [50]. This inhibitory effect is especially apparent for *N*. *gonorrhoeae* strain WHO X (S2 Fig).

Like MTT, NBT at a working concentration of 0.1mg/mL appears slightly yellow before growth (Fig 3A) and turns to a grey purple after growth. However, NBT inhibits *N*. *gonorrhoeae* growth much more than MTT, resulting in no noticeable color change when dye and bacteria are incubated together (Fig 3B). This dye only works reliably as a gonorrhea indicator when added 1 hour before the end of growth (Fig 3C). It also has the least color change when compared to the other dyes studied and did not display strong color change at all in *N*. *gonorrhoeae* strains WHO G, U, V, W, or Y (S3 Fig).

XTT was by far the best tetrazolium dye tested. At a working concentration of 0.1mg/mL, it appeared clear and colorless before growth (Fig 3A) and turned a vibrant orange after growth. It did not appear to inhibit *N*. *gonorrhoeae* growth, in fact, the color was more vibrant when added at the beginning of the 24-hour growth phase (Fig 3B) rather than during the last hour of incubation (Fig 3C) implying XTT may take longer to be reduced than MTT and NBT. XTT was the only dye tested that did not significantly inhibit natural gonococcal growth, making it ideal for clinical diagnostics that aim to minimize sample handling and processes steps since the dye can be pre-loaded with the sample. However, XTT did exhibit some strain variation with poor color change being seen in *N*. *gonorrhoeae* strains WHO U, X, and Z (S4 Fig).

## Discussion

The optimal cell viability media and dye pair for *N*. *gonorrhoeae* low-cost detection should be: 1) highly visible to the naked eye, 2) on the order of cents per test, 3) not inhibit the natural growth, and 4) display minimal strain-to-strain variability. Graver Wade media displayed equivalent growth to Fastidious Broth and was clear and colorless, therefore we selected it for further experimentation with various dyes. *N*. *gonorrhoeae* was able to grow at a pH ranging from 6.8–8.2 but preferred 6.8, since this is considered the cutoff for most pH indicators, the sensitivity would be lacking. Next, we tested resazurin in the form of PrestoBlue and found the color changed dramatically when added after growth and is cost effective (Table 1). However, consistent with research by Schmitt *et al*. [19], we found that resazurin was toxic to *N*. *gonorrhoeae* over time, resulting in poor color change if added prior to growth. MTT was the cheapest option evaluated and displayed robust color change regardless of the growth stage in which it was added but it also showed signs of inhibiting growth. NBT seemed to be the most toxic, apparent by the complete lack of color change when co-incubated for 24 hours. Overall, XTT was the least toxic but the most expensive (Table 1).

**Table 1:** Dye cost comparison.

Indicator	Concentration	Cost for 1mL Culture
**Presto Blue**	10% v/v	$0.06
**MTT**	0.1mg/mL	$0.01
**NBT**	0.1mg/mL	$0.02
**XTT**	0.1mg/mL	$0.19

Cost comparison of PrestoBlue, MTT, NBT, and XTT based on listed working concentrations and prices from ThermoFischer Scientific.

Every strain responded slightly differently to each dye (S1–S4 Figs) which has also been seen with other organisms [51]. Therefore, it is important to test any colorimetric diagnostic on a wide variety of *N*. *gonorrhoeae* strains to ensure accurate results. Our study was constrained to only a single concentration of each dye (1/10 PrestoBlue and 0.1mg/mL MTT, NBT, and XTT) to allow for a comparison of relative toxicity. Further research is required to determine optimum concentrations for each dye that balances strong color change with minimum bacterial growth inhibition. Additionally, MTT, NBT, and XTT were dissolved in water to minimize confounding factors that could limit *N*. *gonorrhoeae* growth, but PBS, DMSO, or another solvent may improve their functionality [52,53]. Overall, these resazurin and tetrazolium-based dyes have potential for simple, low-cost colorimetric readouts of *N*. *gonorrhoeae* viability that could enable a desperately needed low-cost test for antibiotic susceptibility.

This works enables further development of a low-cost or point-of-care susceptibility test for *N*. *gonorrhoeae*, however, some limitations remain. Herein we only tested pure bacterial samples of lab adapted strains and further testing on patient samples is required. Typically, isolation of pure gonococcus from patients requires 24–72 hours of culture on selective media and careful transport to the microbiology lab [54]. Therefore, future research should focus on rapid, effective methods for isolating *N*. *gonorrhoeae* from urine or vaginal swab samples since pH indicators, resazurin dyes, and tetrazolium dyes are all non-specific indicators. However, this also makes a technology developed with these dyes amenable to susceptibility testing of any pathogenic organism, rather than just *N*. *gonorrhoeae*. Future work should also focus on determining minimum inhibitory concentrations of clinically relevant antibiotics for *N*. *gonorrhoeae*, such as ciprofloxacin, ceftriaxone, and azithromycin using XTT as an indicator. While this has never been explored for gonorrhea, XTT determined MIC values have been verified for *Pseudomonas aeruginosa* [55], *Mycobacterium tuberculosis* [56], and *Helicobacter pylori* [57]. Although there is still more work to be done, we hope that the research discussed here provides a starting point to a colorimetric liquid microdilution or a paper microfluidic device susceptibility testing of *N*. *gonorrhoeae*, as has been developed for other organisms [35,58].

## Materials and methods

*N*. *gonorrhoeae* ATCC 49226 and the WHO *N*. *gonorrhoeae* Reference panel [23,24] (CDC & FDA Antibiotic Resistance Isolate Bank. Atlanta, GA). All strains were stored at -80˚C and revived and replated on prewarmed Chocolate Agar (Hardy Diagnostics, USA) at 37˚C and 5% CO_2_ prior to use in all experiments. Graver Wade media was made following standard procedures by Graver and Wade [26] and Fastidious Broth was purchased from Hardy Diagnostics (USA). Bacteria were adjusted to an optical density of 0.1 and diluted 1/20 resulting in ~5x10^6^ CFU/mL starting concentration, as confirmed through serial dilutions on chocolate agar plates.

PrestoBlue Cell Viability Dye was purchased from ThermoFischer Scientific (USA), stored at 4˚C, and diluted to a working concentration of 10% v/v as per manufacturer instructions. MTT and NBT were stored at 4˚C, and 1mg/mL stock concentrations were made prior to each experiment. XTT was stored at -20˚C, and 1mg/mL stock concentrations were made prior to each experiment. MTT, NBT, and XTT were all sterile filtered using a 0.2μm filter and diluted 1/10 for a working concentration of 0.1mg/mL.

## Supporting information

S1 Fig PrestoBlue strain variation. Top: *N*. *gonorrhoeae* strains incubated for 24 hours with 1/10 PrestoBlue added at 23 hours. Bottom: *N*. *gonorrhoeae* strains incubated for 24 hours with 1/10 PrestoBlue added at 0 hours.
(TIF)

S2 Fig MTT strain variation. Top: *N*. *gonorrhoeae* strains incubated for 24 hours with 0.1mg/mL MTT added at 23 hours. Bottom: *N*. *gonorrhoeae* strains incubated for 24 hours with 0.1mg/mL MTT added at 0 hours.
(TIF)

S3 Fig NBT strain variation. Top: *N*. *gonorrhoeae* strains incubated for 24 hours with 0.1mg/mL NBT added at 23 hours. Bottom: *N*. *gonorrhoeae* strains incubated for 24 hours with 0.1mg/mL NBT added at 0 hours.
(TIF)

S4 Fig XTT strain variation. Top: *N*. *gonorrhoeae* strains incubated for 24 hours with 0.1mg/mL XTT added at 23 hours. Bottom: *N*. *gonorrhoeae* strains incubated for 24 hours with 0.1mg/mL XTT added at 0 hours.
(TIF)
