# Supplementary figures and images for: Visible colorimetric growth indicators of Neisseria gonorrhoeae for low-cost diagnostic applications
Source: PLoS One. 2021 Jun 17;16(6):e0252961. doi: 10.1371/journal.pone.0252961 (PMC8211239; doi:10.1371/journal.pone.0252961)

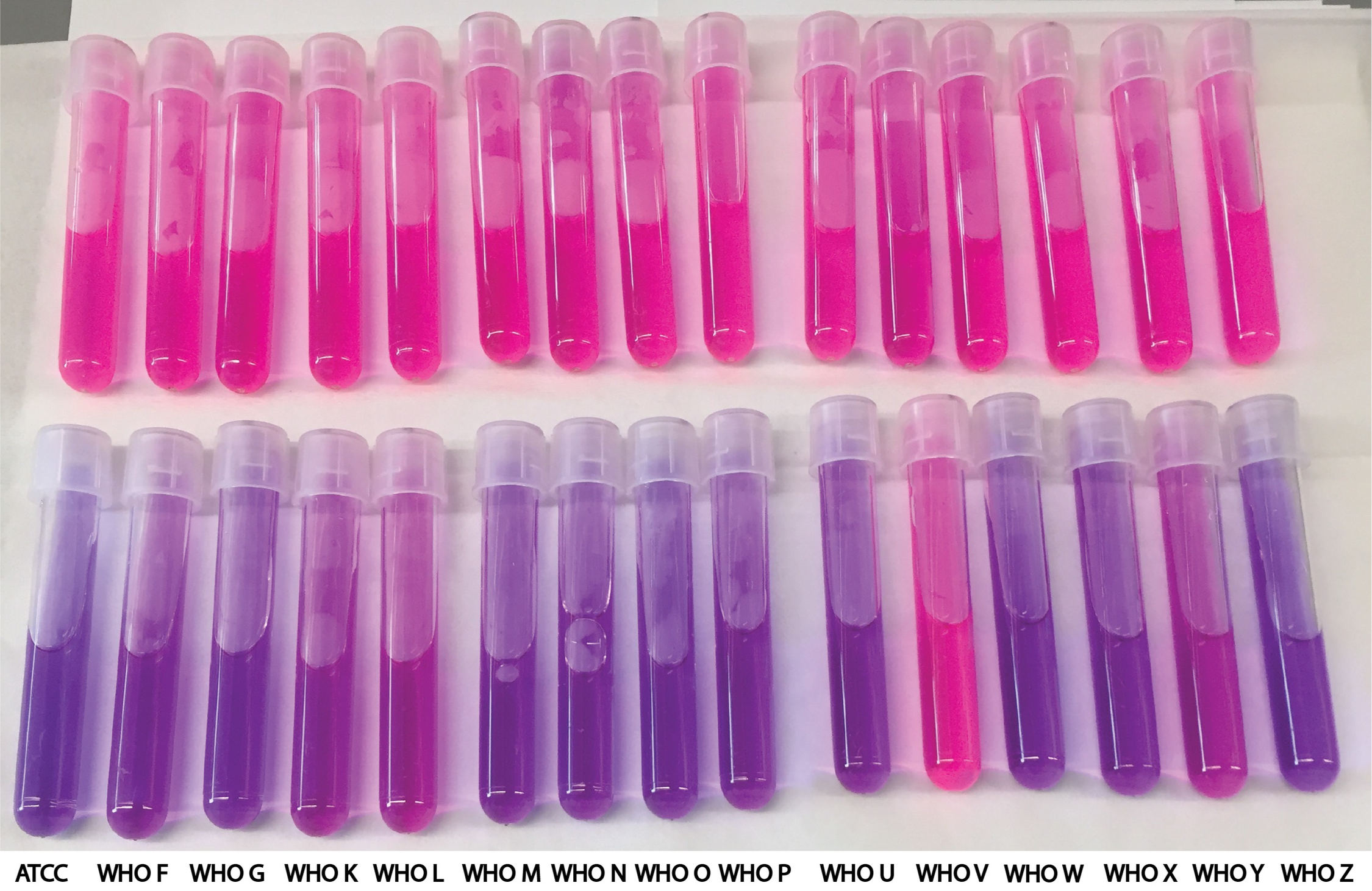

Supplement: S1 Fig — Top: N. gonorrhoeae strains incubated for 24 hours with 1/10 PrestoBlue added at 23 hours. Bottom: N. gonorrhoeae strains incubated for 24 hours with 1/10 PrestoBlue added at 0 hours. (TIF) [file pone.0252961.s001.tif]

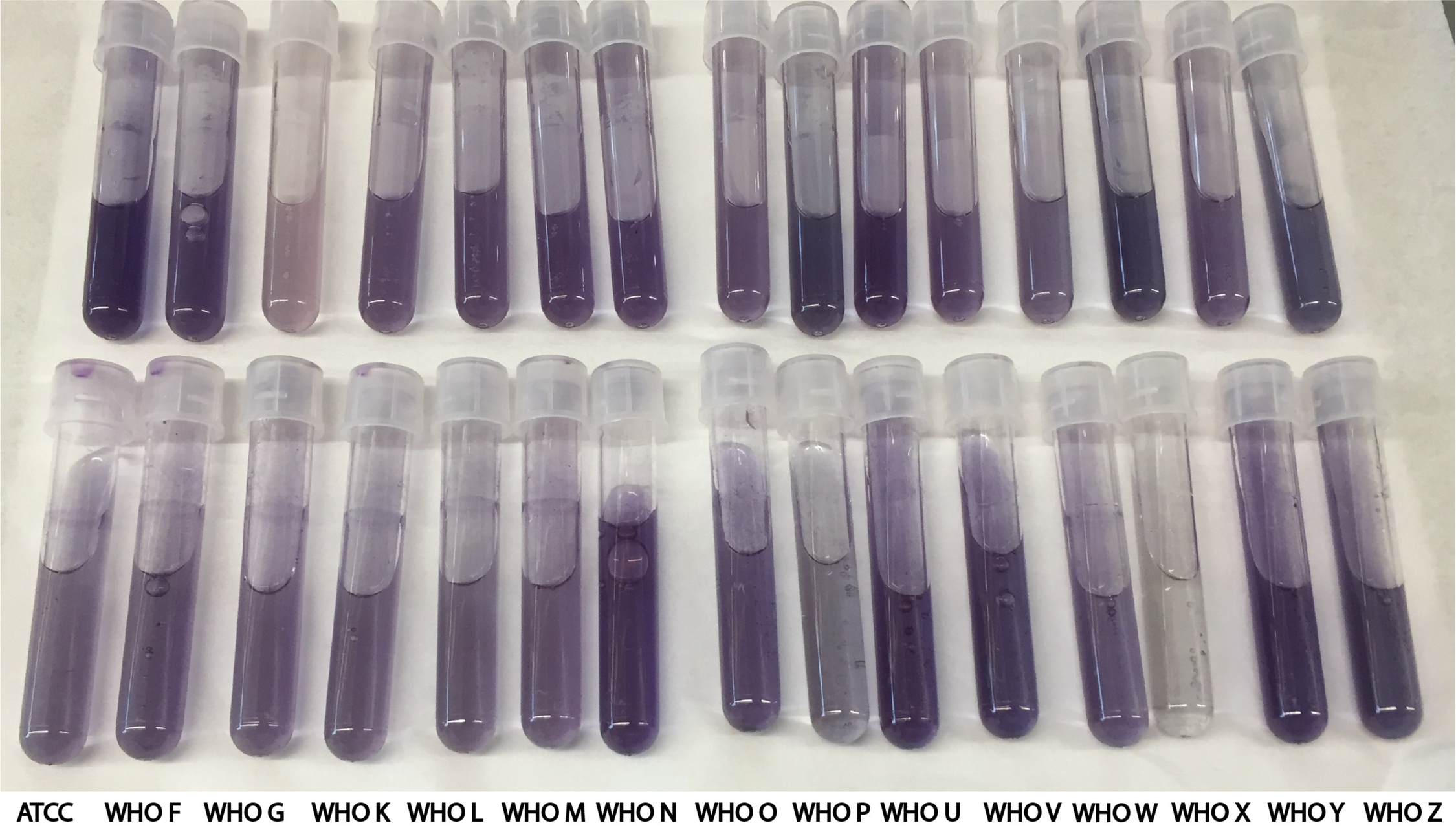

Supplement: S2 Fig — Top: N. gonorrhoeae strains incubated for 24 hours with 0.1mg/mL MTT added at 23 hours. Bottom: N. gonorrhoeae strains incubated for 24 hours with 0.1mg/mL MTT added at 0 hours. (TIF) [file pone.0252961.s002.tif]

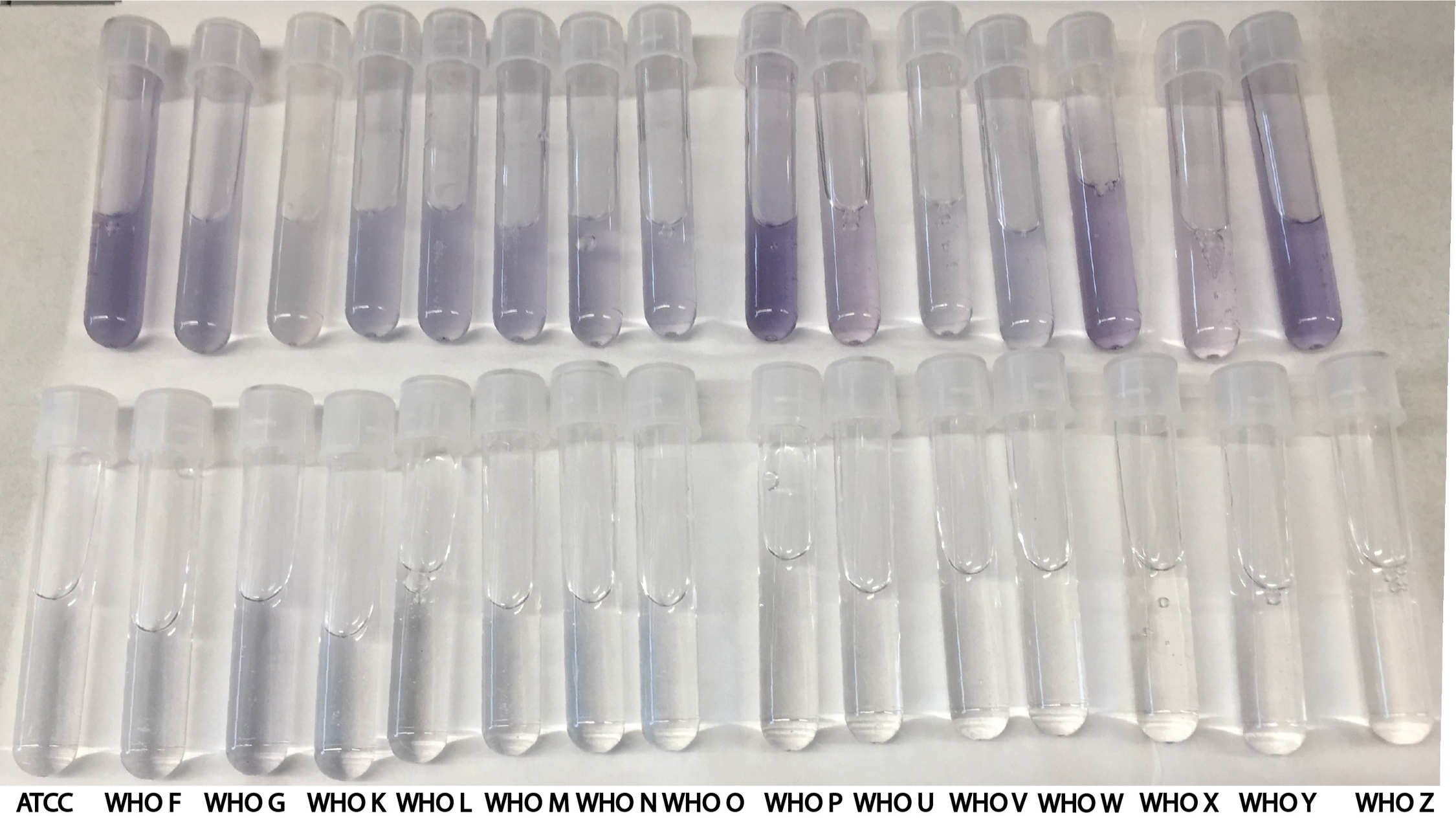

Supplement: S3 Fig — Top: N. gonorrhoeae strains incubated for 24 hours with 0.1mg/mL NBT added at 23 hours. Bottom: N. gonorrhoeae strains incubated for 24 hours with 0.1mg/mL NBT added at 0 hours. (TIF) [file pone.0252961.s003.tif]

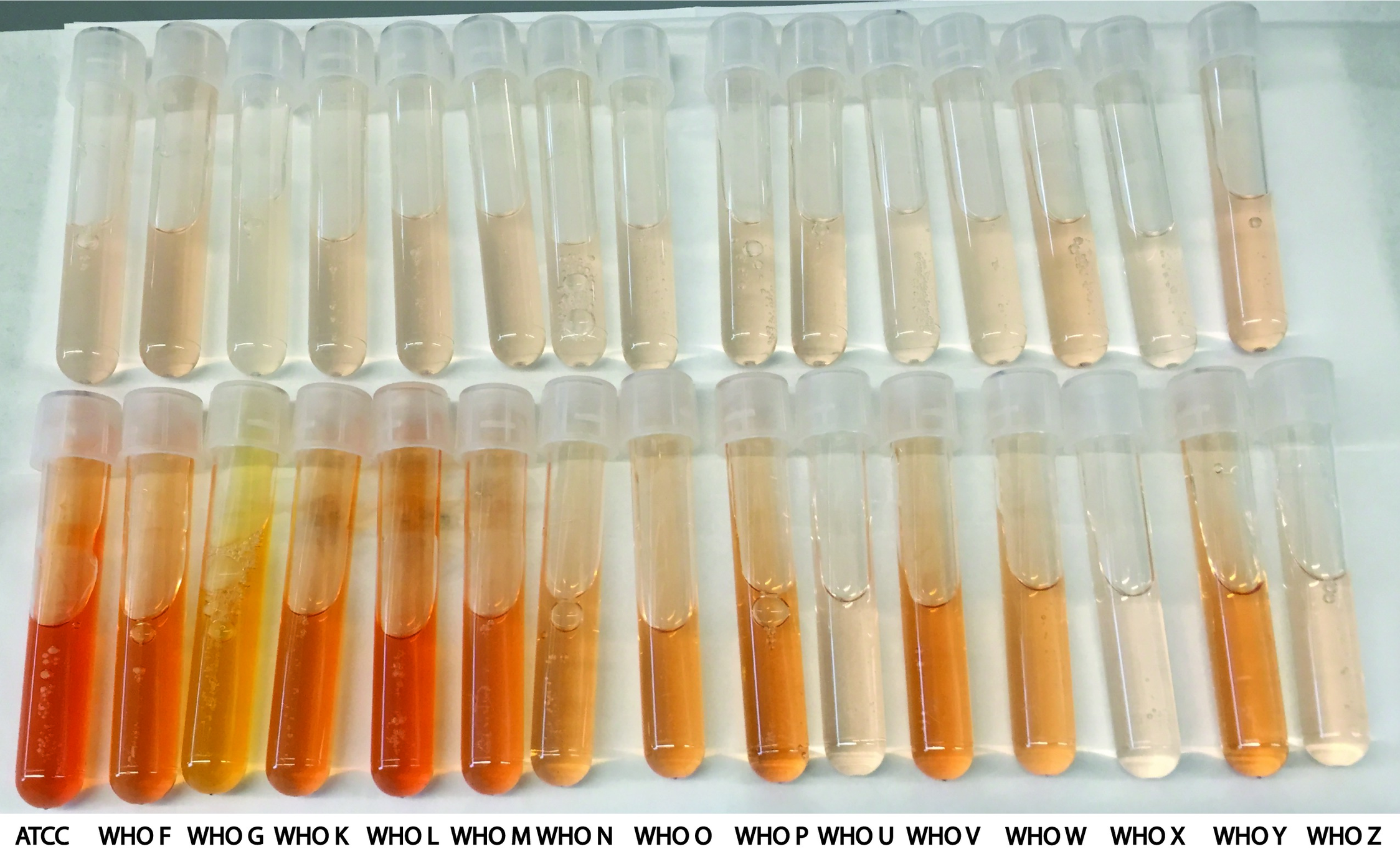

Supplement: S4 Fig — Top: N. gonorrhoeae strains incubated for 24 hours with 0.1mg/mL XTT added at 23 hours. Bottom: N. gonorrhoeae strains incubated for 24 hours with 0.1mg/mL XTT added at 0 hours. (TIF) [file pone.0252961.s004.tif]
